# Supplementary material for: RENGE infers gene regulatory networks using time-series single-cell RNA-seq data with CRISPR perturbations
Source: Commun Biol. 2023 Dec 28;6:1290. doi: 10.1038/s42003-023-05594-4 (PMC10754834; doi:10.1038/s42003-023-05594-4)
Supplement: Supplementary file 1 — Supplementary Information [file 42003_2023_5594_MOESM1_ESM.pdf]

Supplementary Information for: RENGE infers gene regulatory networks using time-series single-cell RNA-seq data with CRISPR perturbations

Supplementary Note 1: Diagonal elements of A

$\mathbf{A}_{i,i}$ ($i = 1, \ldots, G$) represents the concentration-dependent effects from gene i to gene i including self-regulation and degradation, which cannot be estimated separately. Elements of $\mathbf{A}^k$ ($k \geq 2$) include the products of $\mathbf{A}_{i,i}$s. For example, $\mathbf{A}_{i,j} \cdot \mathbf{A}_{i,i}$ ($i \neq j$) represents the strength of the regulation from gene j to gene i multiplied by the strength of degradation or self-regulation of gene i. As only genes i and j are involved, this term should be interpreted as a first-order regulation and not a second-order regulation. Therefore, a more precise explanation for the elements of $\mathbf{A}^k$ ($k \geq 2$) is that they are a mixture of the effects of k'-th order regulation ($k' \leq k$) and degradation.

Supplementary Note 2: Possible future extensions of RENGE

In this study, RENGE was applied to scCRISPR data for hiPSC, a cell type in a steady state. However, GRN inference can also be performed using scCRISPR data for cells in a transient state of dynamics such as differentiating cells under the assumption that the gene regulation $\mathbf{A}$ remains the same during the dynamics. In this situation, with the gene expression data of wild-type and knockout cells sampled at multiple time points, the GRN can be inferred using the same model equation as in this study. Thus, it is possible, for example, to infer the GRN in cells during differentiation.

The inference accuracy of GRNs using our data could be improved as follows: (1) Use of pesudotime. RENGE is a time series model that uses the sampling time of cells as temporal information. Alternatively, we may develop a model that integrates the information of pseudotime, which is estimated from the expression change in each cell [1], as temporal information with a higher time resolution. (2) Use of data from other modalities. The RENGE method can only infer GRNs from transcriptome data.

However, GRN inference methods that utilize the DNA binding motifs of TFs [2, 3], or chromatin accessibility data [4, 5, 3], as well as transcriptome data, have recently been developed and have been proven useful. Therefore, GRN inference methods that integrate time-series transcriptome data following gene perturbations with data from other modalities could improve inference accuracy.

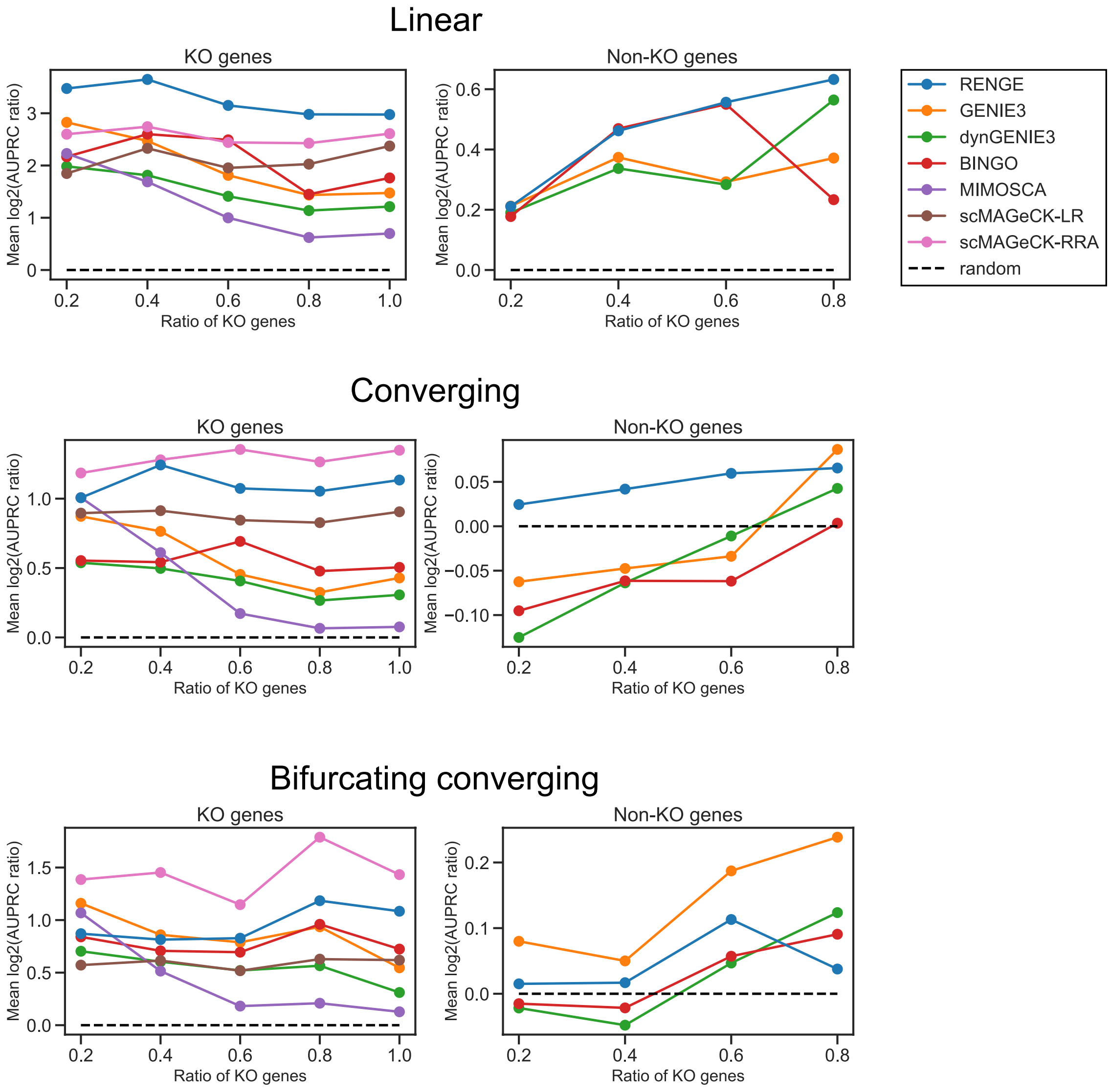

Supplementary Figure 1: **Benchmark results from the simulated data sets for each network generated using the three backbones defined in dyngen with GRN filtering.** GRNs were filtered based on the amount of expression variation caused by single-gene KO (MIMOSCA score). Horizontal axis: knocked-out gene ratio in the GRNs.

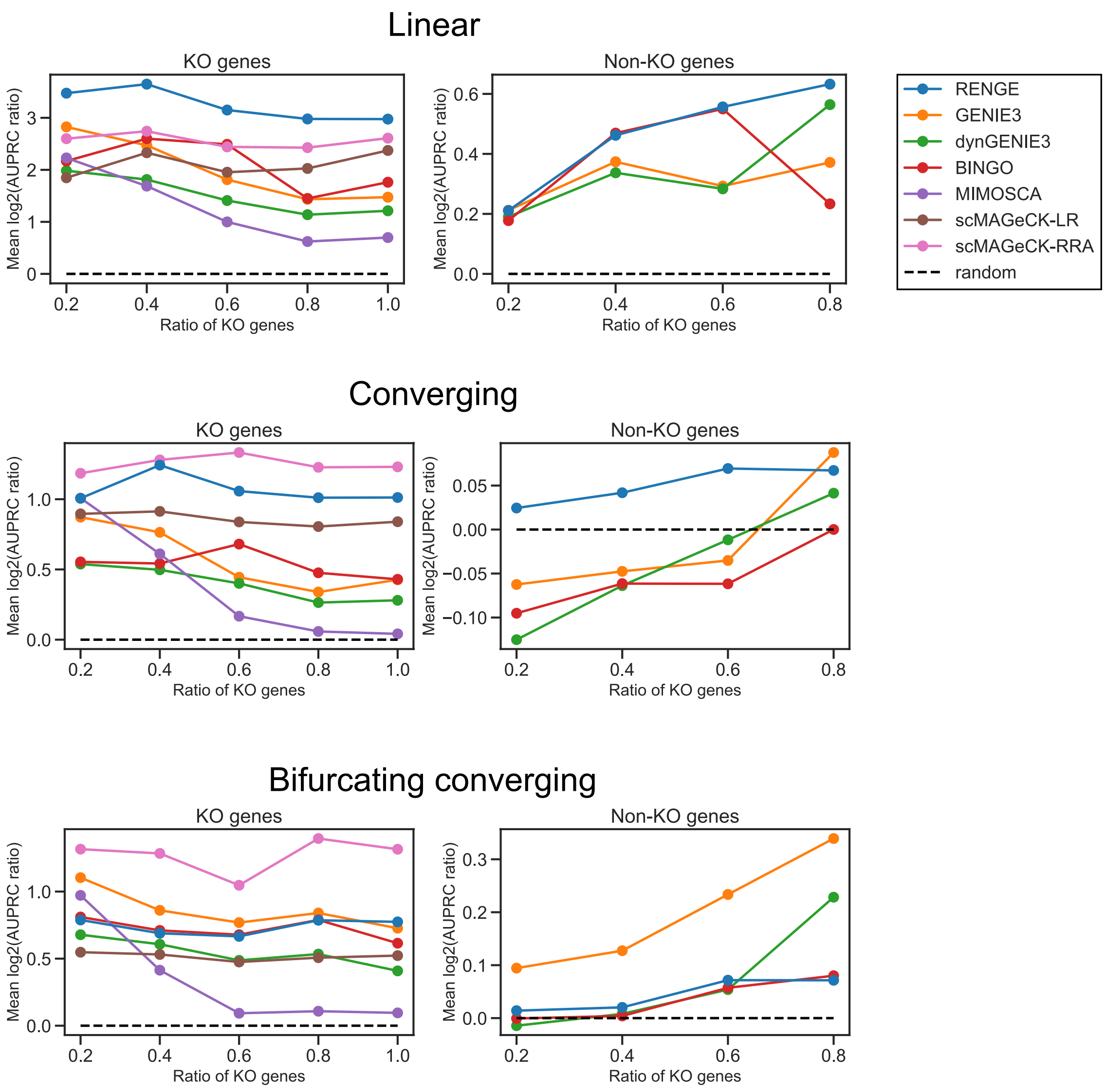

Supplementary Figure 2: **Benchmark results from the simulated data sets for each network generated using the three backbones defined in dyngen without GRN filtering.** All the 750 GRNs were used without filtering. Horizontal axis: knocked-out gene ratio in the GRNs.

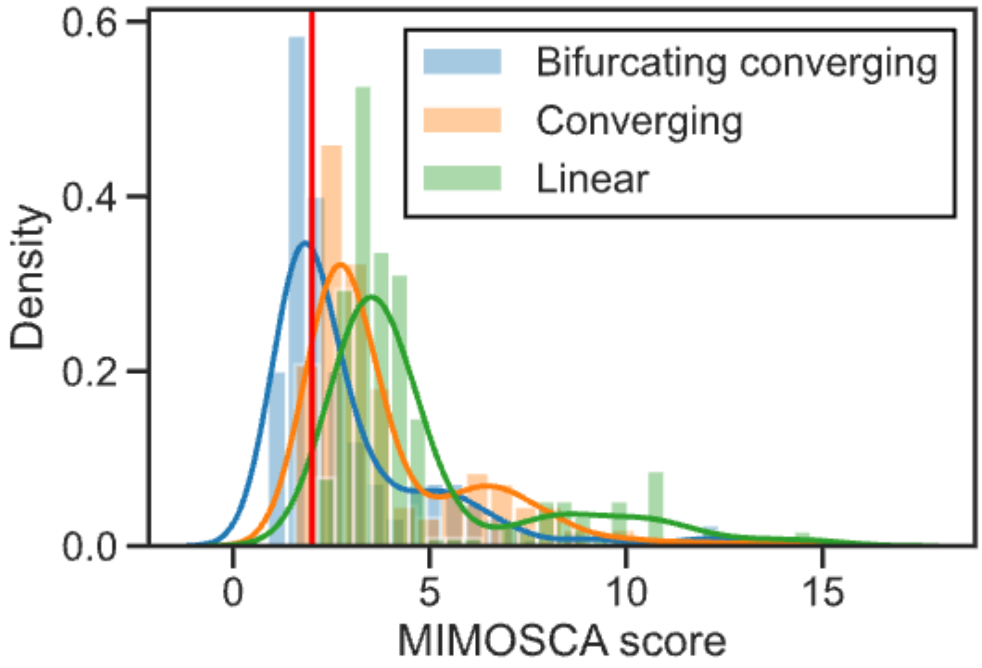

Supplementary Figure 3: **Distribution showing MIMOSCA score for each backbone.** The red vertical line indicates the filtering threshold, MIMOSCA_score = 2.

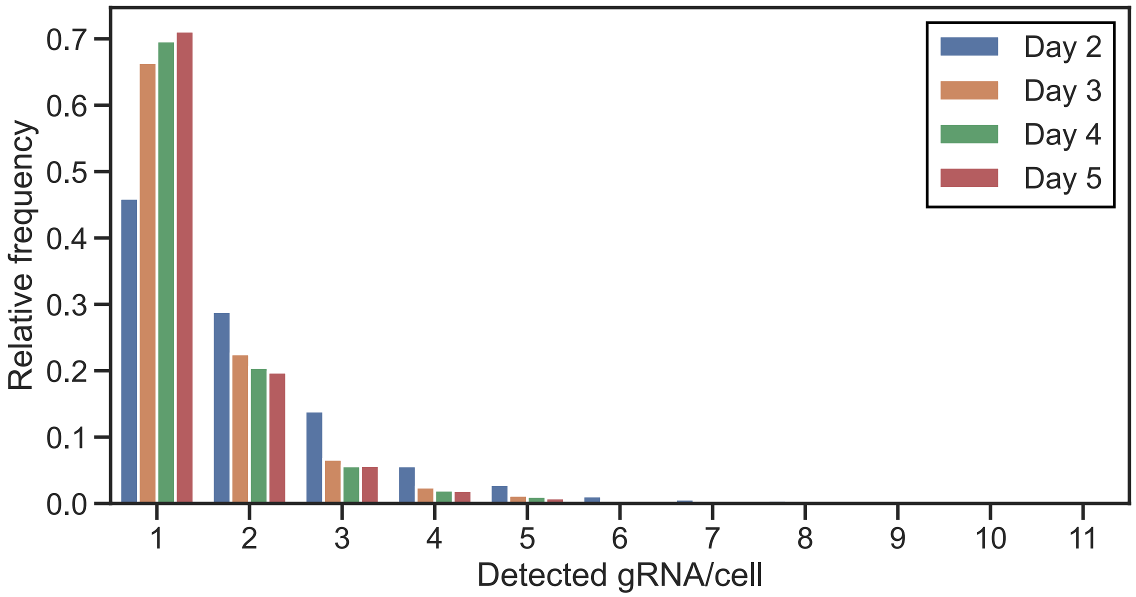

Supplementary Figure 4: **Distribution showing the number of gRNAs detected each day per hiPSC.**

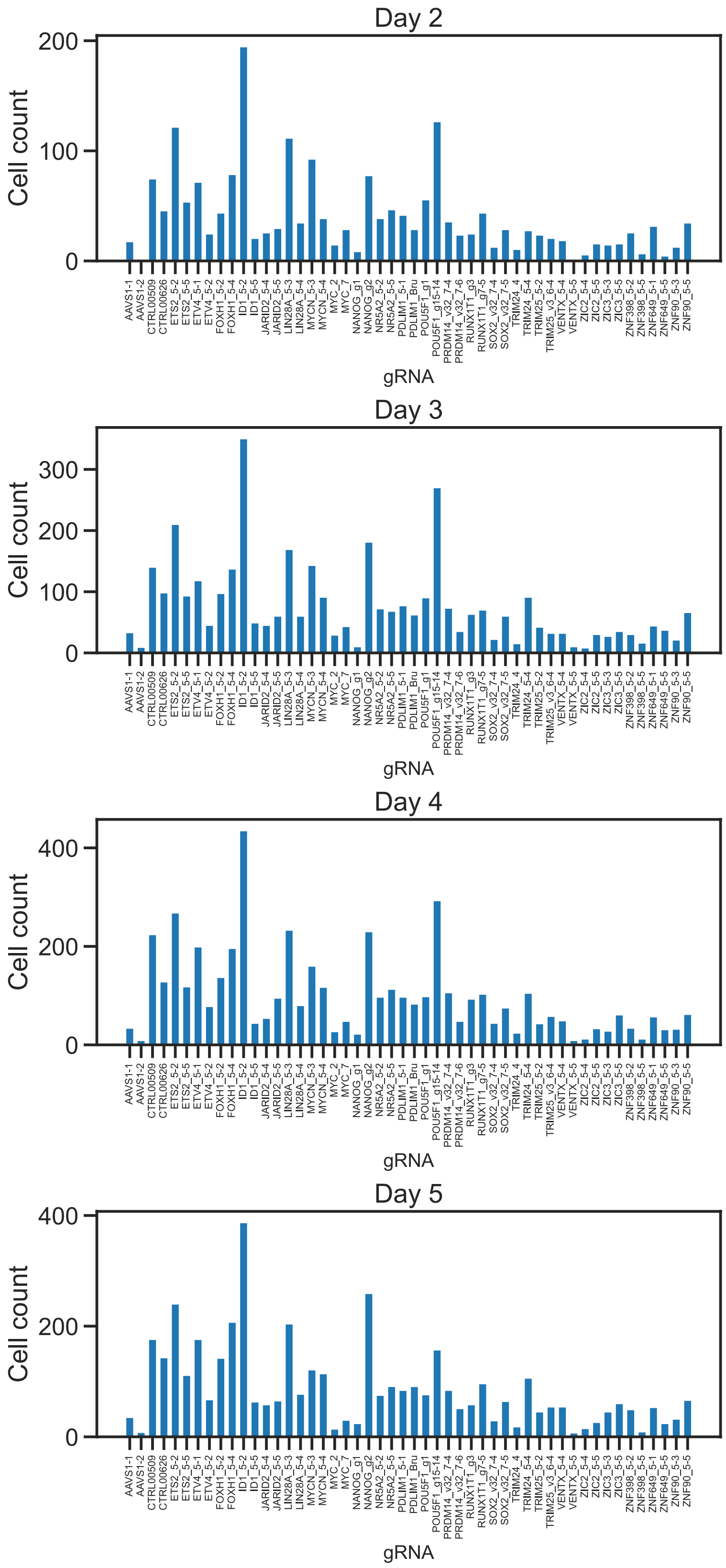

Supplementary Figure 5: **Number of cells in which each gRNA was detected.** Only cells bearing a single gRNA were counted.

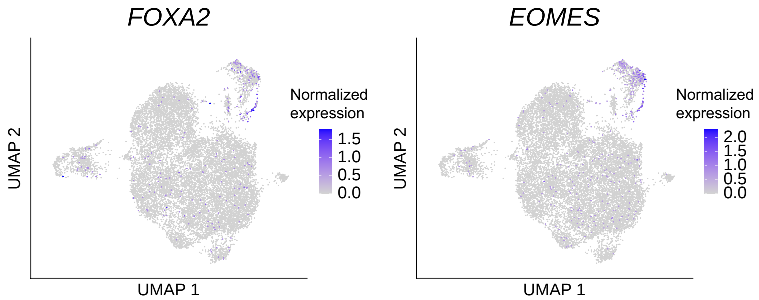

Supplementary Figure 6: **Marker expression on the UMAP plot.** Colors indicate the expression of *FOXA2* or *EOMES* (mesoendoderm markers).

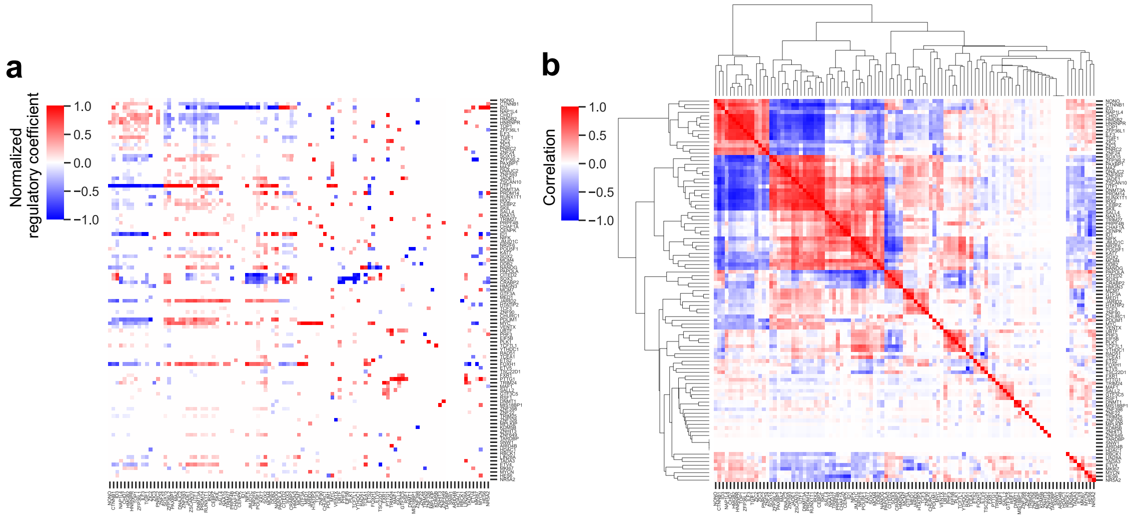

Supplementary Figure 7: **Regulatory coefficients and regulatory correlations in hiPSCs.** **a**, Regulatory coefficients between 103 genes, showing the strength of regulation from the gene in each column to the gene in each row. The regulatory coefficients with q-value ≥ 0.01 were set to 0, while the others were normalized so that the maximum absolute value of the regulatory coefficients in each column was 1. **b**, Regulatory correlations between 103 genes. A regulatory correlation is the correlation coefficient between the regulatory coefficients of the gene in each column and the gene in each row.

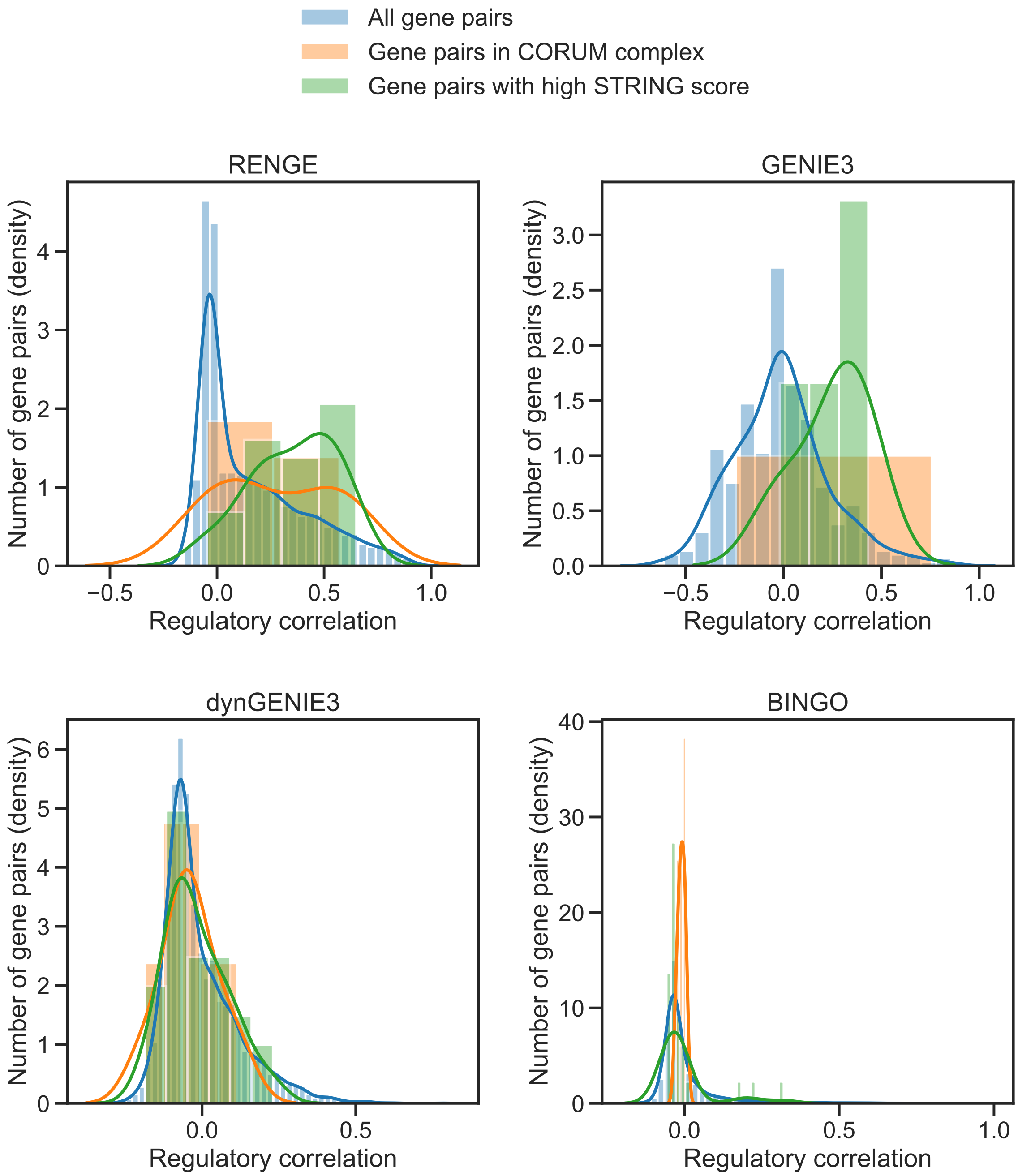

Supplementary Figure 8: **Distributions showing the regulatory correlations among all gene pairs estimated using each method.** Distribution of the regulatory correlations among all possible gene pairs compared with those in the protein complexes obtained from CORUM3.0 and those with high STRING scores. For each method, 1354 regulations, the number of regulations with q-value < 0.01 in RENGE, were selected in order of confidence level, and the regulatory coefficients for the remaining regulations were set to 0. Regulatory correlations for RENGE were calculated using the absolute value of the regulatory coefficients ignoring the sign for consistency with other methods, which only infer regulatory strength without the sign. Gene pairs with correlation coefficients that could not be calculated due to no inferred regulation from the gene, are not shown. For GENIE3, the kernel density estimate for gene pairs in the CORUM complex is not shown as only one gene pair was included in the CORUM complex.

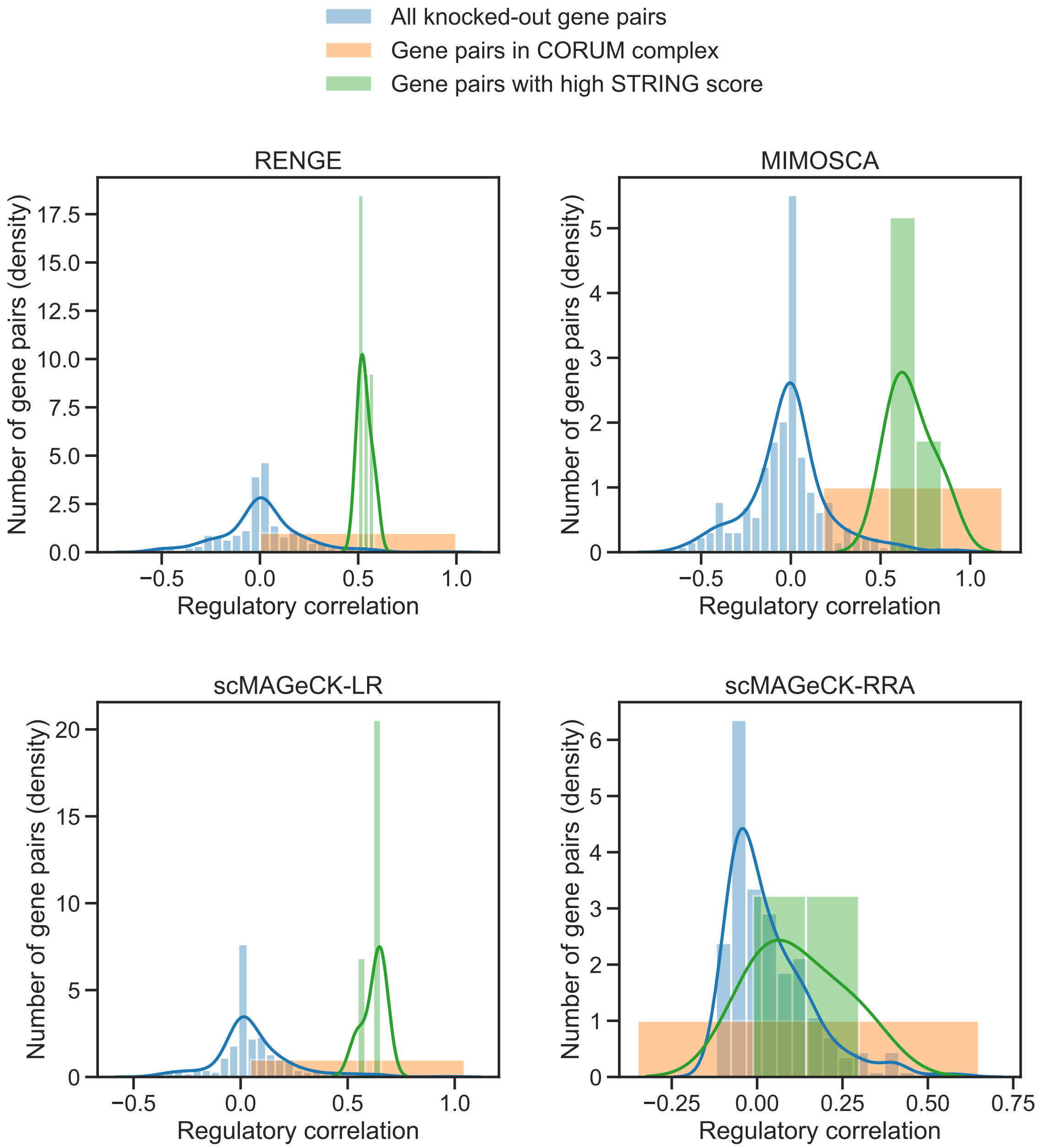

Supplementary Figure 9: **Distributions showing the regulatory correlations among the knocked-out genes that were estimated using each method.** Distribution of the regulatory correlations among all possible pairs in the knocked-out genes when compared with those in the protein complexes obtained from CORUM3.0 and those with high STRING scores. For each method, 346 regulations, the number of regulations with q-value < 0.01 in RENGE, were selected in order of confidence level of the regulations, and the regulatory coefficients or q-values for the remaining regulations were set to 0 or 1, respectively. Gene pairs with correlation coefficients that could not be calculated due to no inferred regulation from the gene, are not shown. For all four methods, kernel density estimates for gene pairs in the CORUM complex are not shown as only one gene pair was included in the CORUM complex.

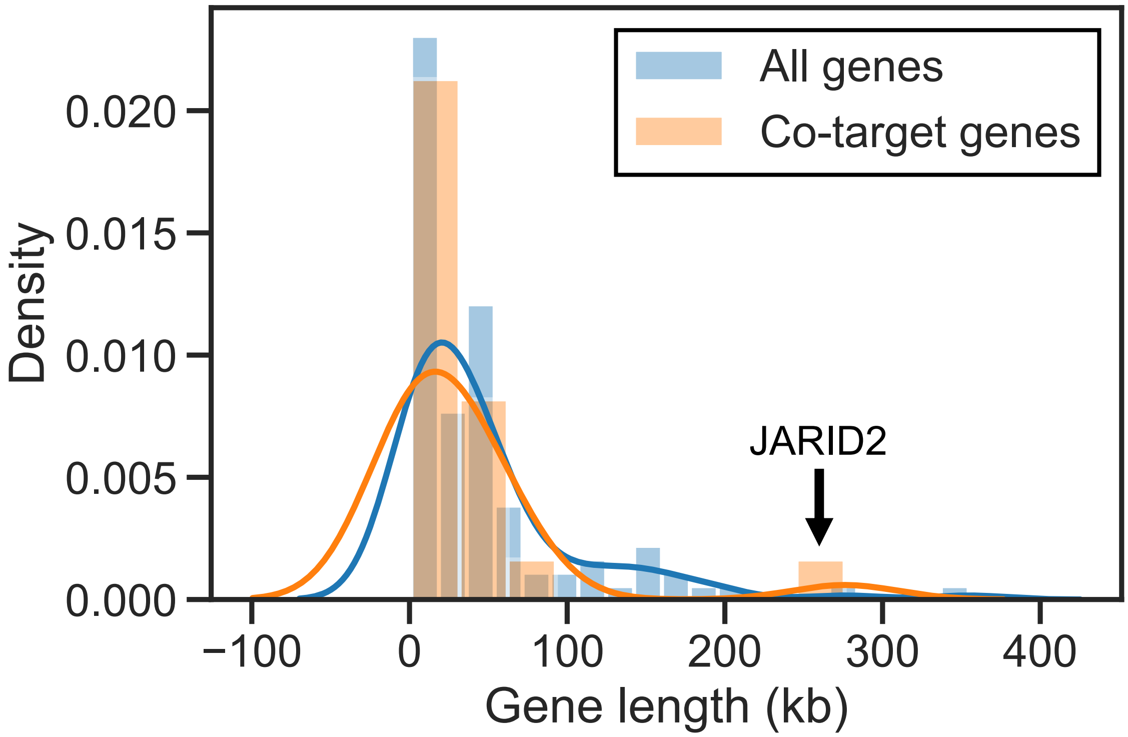

Supplementary Figure 10: **Distribution showing the length of all genes in the GRN versus co-target genes of CHD7 and TOP1.** Co-target genes are those that are regulated by both CHD7 and TOP1 (FDR < 0.01). *JARID2*, the longest gene in the co-target genes is identified by an arrow.

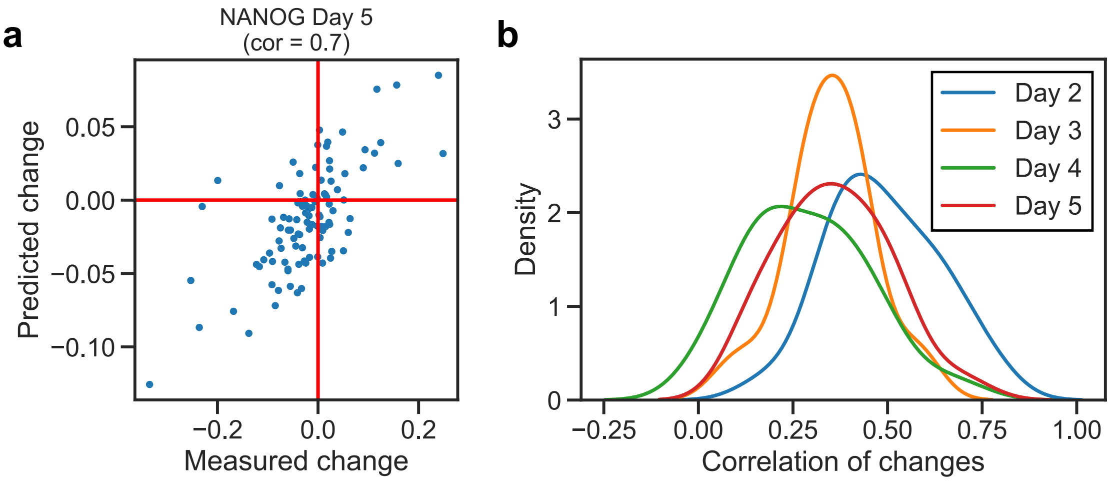

Supplementary Figure 11: **Prediction of expression changes by knockout using RENGE. a**, Relationship between predicted and measured expression changes on day 5 when *NANOG* is knocked out. Each point corresponds to a gene in the network. The horizontal and vertical axes indicate the experimentally measured and predicted changes in expression for each gene when *NANOG* is knocked out, respectively. The prediction was made using the RENGE model learned using data without the *NANOG* knockout. **b**, Distribution of the correlation coefficients between the measured expression changes for each gene and the expression changes predicted by RENGE when each of the 23 genes were knocked out. The RENGE model which was trained without the knockout data for gene i was used to predict the expression changes for each gene when gene i was knocked out.

Supplementary Table 1: gRNA sequences used in the single-cell CRISPR experiment

Gene symbol	sgRNA ID	Guide sequence
Safe-cutter control	AAVS1-1	GTCCCTAGTGGCCCCACTG
Safe-cutter control	AAVS1-2	GGGCCACTAGGGACAGGAT
Non-cutter control	CTRL00509	CGAATTCACTCCCTTAGCG
Non-cutter control	CTRL00626	ACGCCCAGACTGTTAGCGC
ETS2	ETS2_5-2	TGCAGAGGTTCGGCATGAA
ETS2	ETS2_5-5	CACAGAATTACCCCAAAGG
ETV4	ETV4_5-1	GGGCACCCCGGCGCTGGTA
ETV4	ETV4_5-2	GGTCGGTGCATTTCGAGAG
FOXH1	FOXH1_5-2	GCTGCACGGCCGGCCATAC
FOXH1	FOXH1_5-4	CGGTTTCAGATCATCCGTC
ID1	ID1_5-2	GGCCATCTCGCGCTGCGCC

ID1	ID1_5-5	TACATCAGGGACCTTCAGT
JARID2	JARID2_5-4	ATCGAGTCGGTCCGCGCTC
JARID2	JARID2_5-5	GGTGATCCCCCCTCCGGAC
LIN28A	LIN28A_5-3	TGTCCATGACCGCCCGCGC
LIN28A	LIN28A_5-4	GAGTAAGCTGCACATGGAA
MYC	MYC_2	TATTTCTACTGCGACGAGG
MYC	MYC_7	GGGTCGATGCACTCTGAGG
MYCN	MYCN_5-3	TCGCGCTTGTTCACGGGAA
MYCN	MYCN_5-4	GGTATTGCCGCCCCAGCCG
NANOG	NANOG_g1	CCTCAGCTACAAACAGGT
NANOG	NANOG_g2	GAGAAGAGTGTCGCAAAAA
NR5A2	NR5A2_5-2	AGTCCATTGGCTCGGATGA
NR5A2	NR5A2_5-5	GATGGCCCGGCTAGGAAAG
PDLIM1	PDLIM1_5-1	GGCAGCAGTCTTTGACTCC
PDLIM1	PDLIM1_Bru	ATGAGCAAGAACTTACCTG
POU5F1	POU5F1_g1	GTGGGTTTCGGGCACTGC
POU5F1	POU5F1_g15-14	CTTGCAGGTGGTCCGAGTG
PRDM14	PRDM14_v32_7-4	GCTCGGTTCCAGTTCACGG
PRDM14	PRDM14_v32_7-6	GCAGCAGCCACGAGTACGC
RUNX1T1	RUNX1T1 g3	GAGTTCGCACCCTCGTTCT
RUNX1T1	RUNX1T1 g7-5	TACCACTAGTCCCAGAACG
SOX2	SOX2_v32_7-4	GCGGGCGTGAACCAGCGCA
SOX2	SOX2_v32_7-5	TTATAAATACCGGCCCCGG
TRIM24	TRIM24_4	TTTGAGCTCACCAGTGGGA
TRIM24	TRIM24_5-4	TTGCATAAGTTTCATGCGA
TRIM25	TRIM25_5-2	GGCGCAACAGGTCGCGAAC
TRIM25	TRIM25_v3_6-4	AACACGGTGCTGTGCAACG
VENTX	VENTX_5-4	TCTATGTCAGGGTTGAGTA
VENTX	VENTX_5-5	GACGTTGAGTAGAAAGCTG
ZIC2	ZIC2_5-4	CATATTCATGGGGCCGTAC
ZIC2	ZIC2_5-5	GGGATTGCTCAGTTGCTCG
ZIC3	ZIC3_5-3	TCGCGCGTTGAGTTGAAGG

ZIC3	ZIC3_5-5	ACTGAGCGCCGGCCGCGTA
ZNF398	ZNF398_5-2	CGTCACTCTCCTTGGACTC
ZNF398	ZNF398_5-5	ACACCTGCTCGCCAACTGG
ZNF649	ZNF649_5-1	AATCATGAACAAATGCCTA
ZNF649	ZNF649_5-5	CACAAACAGGGTATCAAGC
ZNF90	ZNF90_5-3	TTATGTACATAAAGGATCG
ZNF90	ZNF90_5-5	TTATGTTTATAAAGGAGTG

Supplementary Table 2: List of the oligonucleotides used to prepare the gRNA sequencing library

GuideForward	5'-AATGATACGGCGACCACCGAGATCTACACTCTTTCCCTACACGACGCTCTTCCGATCT-3'
GuideReverse-R1	5'-CAAGCAGAAGACGGCATACGAGATAGGAGTCCGTCTCGTGGGCTCGGAGATGTGTATAAGAGACAGCGGACTAGCCttatttaaacttgctatgc-3'
GuideReverse-R2	5'-CAAGCAGAAGACGGCATACGAGATCATGCCTAGTCTCGTGGGCTCGGAGATGTGTATAAGAGACAGCGGACTAGCCttatttaaacttgctatgc-3'
GuideReverse-R3	5'-CAAGCAGAAGACGGCATACGAGATGTAGAGAGGTCTCGTGGGCTCGGAGATGTGTATAAGAGACAGCGGACTAGCCttatttaaacttgctatgc-3'
GuideReverse-R4	5'-CAAGCAGAAGACGGCATACGAGATCAGCCTCGGTCTCGTGGGCTCGGAGATGTGTATAAGAGACAGCGGACTAGCCttatttaaacttgctatgc-3'

Supplementary Table 3: Genes with ChIP-seq data obtained from the ChIP-Atlas.

Gene with ChIP-seq	Knock out
FOXH1	Yes
JARID2	Yes
MYC	Yes
NANOG	Yes
NR5A2	Yes
POU5F1	Yes
PRDM14	Yes
SOX2	Yes
ZNF398	Yes
CHD7	No
CTNNB1	No
DNMT1	No
DNMT3A	No
KDM5B	No
MED1	No
SALL4	No
TCF3	No
TCF7L1	No
UBTF	No

Supplementary Table 4: Correlation coefficients between the confidence of inferred regulation and ChIP-seq $-10 * \log_{10}$(MACS2 q-value)

	Regulations by KO genes	Regulations by non-KO genes
RENGE	0.292	**0.290**
GENIE3	**0.390**	-0.048
dynGENIE3	0.123	0.077
BINGO	0.025	0.048
MIMOSCA	0.212	-
scMAGeCK-LR	0.251	-
scMAGeCK-RRA	0.049	-

Supplementary Table 5: Correlation coefficients between the measured and predicted expression changes by RENGE for each day when each of the 23 genes were knocked out.

KO gene	Day 2	Day 3	Day 4	Day 5
ETS2	0.78	0.42	0.44	0.39
ETV4	0.43	0.08	0.18	0.13
FOXH1	0.46	0.37	0.21	0.20
ID1	0.51	0.42	0.30	0.38
JARID2	0.51	0.30	0.14	0.30
LIN28A	0.51	0.34	0.18	0.35
MYC	0.62	0.27	0.17	0.15
MYCN	0.73	0.59	0.34	0.25
NANOG	0.56	0.34	0.67	0.70
NR5A2	0.62	0.56	0.34	0.52
PDLIM1	0.18	0.14	0.06	0.29
POU5F1	0.67	0.29	0.50	0.46
PRDM14	0.33	0.45	0.50	0.56
RUNX1T1	0.34	0.27	0.22	0.39
SOX2	0.41	0.35	0.44	0.52
TRIM24	0.59	0.41	0.21	0.50
TRIM25	0.34	0.35	0.34	0.42
VENTX	0.42	0.29	0.36	0.26
ZIC2	0.46	0.25	0.09	0.14
ZIC3	0.40	0.31	0.01	0.24
ZNF398	0.37	0.41	0.15	0.36
ZNF649	0.35	0.42	0.32	0.46
ZNF90	0.65	0.42	0.41	0.31

Supplementary Table 6: dyngen parameters used for the benchmark.

Parameter	Value
total_time	simtime_from_backbone(backbone) + 100
num_timepoints	ceiling(total_time / 100 × 4)
timepoint (of KO)	(total_time - 100) / total_time
multiplier	rbinom(num_simulations, 1, 0.3)
pct_between	0.1
num_cells	25 × num_timepoints
num_tfs	100
num_targets	0
num_hks	0
n_census	10 × num_cells
census_interval	floor((total_time × num_simulations) / n_census)
tau	0.5

Supplementary References

[1] Cao, J. *et al.* The single-cell transcriptional landscape of mammalian organogenesis. *Nature* **566**, 496–502 (2019).

[2] Aibar, S. *et al.* Scenic: single-cell regulatory network inference and clustering. *Nature methods* **14**, 1083–1086 (2017).

[3] González-Blas, C. B. *et al.* SCENIC+: single-cell multiomic inference of enhancers and gene regulatory networks. *BioRxiv* (2022).

[4] Kamimoto, K., Hoffmann, C. M. & Morris, S. A. Celloracle: Dissecting cell identity via network inference and in silico gene perturbation. *BioRxiv* (2020).

[5] Zhang, L., Zhang, J. & Nie, Q. Direct-net: An efficient method to discover cis-regulatory elements and construct regulatory networks from single-cell multiomics data. *Science Advances* **8**, eabl7393 (2022).
