# Supplementary material for: RENGE infers gene regulatory networks using time-series single-cell RNA-seq data with CRISPR perturbations
Source: Commun Biol. 2023 Dec 28;6:1290. doi: 10.1038/s42003-023-05594-4 (PMC10754834; doi:10.1038/s42003-023-05594-4)
Supplement: Supplementary file 2 — Description of additional supplementary files [file 42003_2023_5594_MOESM2_ESM.docx]

Description of Additional Supplementary Files

**File name:** Supplementary Data 1

**Description:** The Excel file for Supplementary Tables 1-2.

**File name:** Supplementary Data 2

**Description:** The source data behind the graphs in the paper
